# The hidden cause of chronic finger pain: Glomus Tumor – A Case Report

**DOI:** 10.25122/jml-2018-0060

**Published:** 2019

**Authors:** Anca Bordianu, Dragos Zamfirescu

**Affiliations:** 1. Bagdasar-Arseni Emergency Hospital, Plastic and Reconstructive Department, Carol Davila University of Medicine and Pharmacy, Bucharest, Romania; 2. Zetta Clinic, Plastic and Reconstructive Surgery Department, Bucharest, Romania

**Keywords:** glomus tumor, finger, pathology, hand surgery, pain

## Abstract

Glomus tumors are frequently associated with pain, tenderness and cold sensitivity.

We report the diagnosis and successful surgical management of a case of a classic glomus tumor in a young woman.

The clinical diagnosis was made on the basis of medical history and MRI findings. The lesion was excised via a dorsolateral subungual approach, leading to the complete resolution of symptoms. Histology confirmed the lesion to be a glomus tumor.

Glomus tumors are painful subungual lesions. They produce a throbbing or lancinating local discomfort, cold-sensitivity, and severe pain following minor trauma. The diagnosis is confirmed by histology, but the clinical diagnosis is highly suggestive. Complete excision will usually relieve pain. Recurrence is common following incomplete resection.

## Introduction

Glomus tumors are rare, benign perivascular hamartomas of the glomus apparatus. In rare cases, large visceral lesions may be malignant [1].

Glomus tumors are usually under one centimeter in size and consist histologically of glomus bodies. They present as a faint, blue-red subungual papule associated with a classic triad of symptoms: local sensitivity, pain with cold exposure, and severe pain following minor trauma [2].

Glomus tumors develop from modified glomus cells which are specialized smooth muscle cells that function as chemoreceptors [3]. The normal function of glomus cells is to regulate blood flow in capillaries in response to changes in temperature.

Glomus tumors occur most frequently on the hand, especially in the subungual area, where glomus bodies are in high concentration. Glomus tumors may also occur in the lung, stomach, pancreas, liver, gastrointestinal or genitourinary tract [3, 4].

They are rarely malignant. Those that are cancerous tend to be large (over 2 centimeters, profound, and visceral. Giant intravenous glomus tumors have been reported [5].

Glomus tumors grow slowly and can only be detected by MRI years following the first appearance of the symptoms [6,7].

Subungual glomus tumors mostly occur in female patients [8], while for tumors located in other areas of the body, there is no sex predilection [3].

Complete excision is essential in the prevention of recurrence [9], and the only solution to relieve pain, given the fact that anti-inflammatory drugs have little or no effect [10].

Removal of the tumor is performed in a bloodless field, with tourniquet application, under loupe magnification [11]. A bloody field can preclude complete excision and lead to recurrence [12]. When bone is involved, the bony portion of the lesion may either be excised or removed by curettage. En bloc resection is only necessary in the case of malignant tumors.

The surgical approach depends on the location of the lesion. For central subungual glomus tumors removal of the nail is often necessary [13, 14]. The diagnosis is confirmed by histology demonstrating capillaries lined with glomus cells. Glomus tumors are immunoreactive for smooth muscle actin and CD34 [3].

The best chance for cure is at the initial surgery and meticulous preparation, a bloodless field being imperative for tumor removal. In the event that the surgeon does not feel confident that the lesion can be “shelled out,” a peripheral and deep curettage may improve the cure rate.

## Materials and Methods

A 33-year-old female surgeon reported intermittent episodes of severe pain of the dorsolateral side of the distal phalanx of the left thumb.

The onset of symptoms was eight years prior to presentation, and discomfort was worsening in frequency and severity. The patient also reported substantial cold sensitivity. Minor trauma to the thumb produced severe pain.

**Figure 1:**
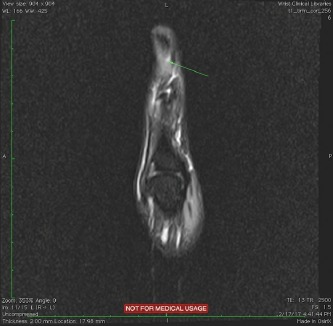
MRI appearance of the tumor – radial side view of the thumb

**Figure 2:**
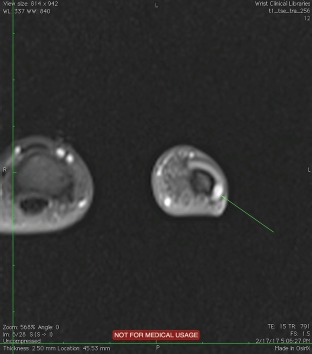
Figure 2: MRI appearance of the tumor – thumb section view

A subungual glomus tumor was suspected. An MRI confirmed the presence of the lesion and its precise location laterally toward the nail fold. (Figure 1 and 2) [15].

Excision was performed with local anesthesia under tourniquet control.

The tumor was excised using a lateral subperiosteal approach without removing the nail plate. The lesion was identified and excised sharply. Where the tumor had depressed the bone of the distal phalanx, curettage was employed to ferret out any microscopic foci of disease. The biopsy specimen was sent to the pathology laboratory.

Following surgery, the patient reported immediate pain relief, and there were no postoperative complications.

## Results

The excised lesion was a solitary, well defined, unencapsulated 3 mm mass of dark red tissue containing small nerve fibers.

**Figure 3:**
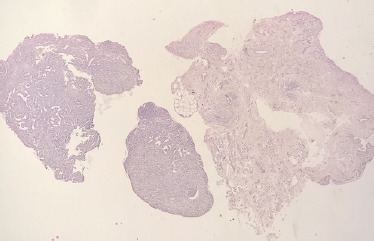
Typical histological appearance of the glomus tumor

Histologically, the tumor was composed of three typical components: glomus cells, smooth muscle cells, small vascular structures (Figure 3).

**Figure 4:**
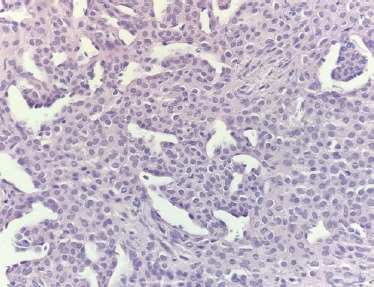
Hematoxylin and eosin (HE) staining demonstrates dense perivascular proliferation with round and oval cells

The tumor mass was composed mainly of monomorphic, round to oval cells with eosinophilic cytoplasm and oval nuclei. The cells were arranged in cords and sheets with festoons, frequently forming perivascular “sleeves” around capillaries. Mitoses were absent (Figure 4).

**Figure 5:**
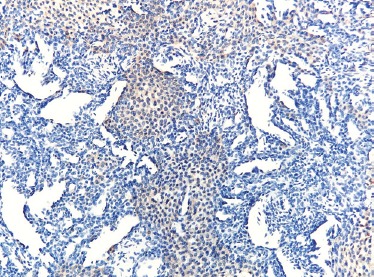
Smooth muscle actin (SMA) positivity in some tumor cells

**Figure 6:**
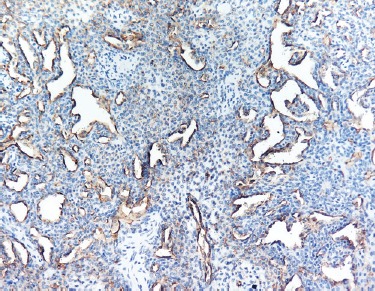
CD34 immunostaining highlighting vessels

**Figure 7:**
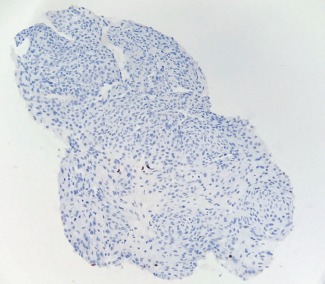
Ki67, Index of cell proliferation or mitosis

Immunohistochemical staining demonstrated positivity for smooth muscle actin (SMA) (Figure 5). CD34 immunostaining highlighted the vascular component (Figure 6). KI-67 (index of proliferation) was found positive in approximatively 2% of the tumor cells (Figure 7). The tumor stained negative for keratins and desmin.

The patient had an unremarkable postoperative course. One year postoperatively she noted no nail deformity, no recurrence and was symptom-free.

## Discussions

Glomus tumors are rare, small, benign neoplasms most commonly noted on the hand, especially the fingers. Peripheral tumors are uniformly benign, but they can cause disabling pain and tenderness.

Subungual glomus tumors occur most commonly in female patients.

Glomus tumors are difficult to detect, due to their small size, slow growth rate, and late detection.

Diagnosis is suggested by a triad of symptoms: local sensitivity, pain associated with the cold environment, and severe pain following minor trauma.

Complete excision is crucial in the prevention of recurrence and resolution of symptoms. MRI is effective in locating the tumor and delineating its extent prior to surgery.

A bloodless field is essential to allow for meticulous removal of the tumor. Periungual glomus tumors will frequently depress the bone of the distal phalanx and may be adherent within the periosteum. When this occurs, as in our case, meticulous curettage of the bone may prevent recurrence.

## Conclusions

Periungual glomus tumors reliably diagnosed on clinical presentation as a small tumor with disproportionate symptoms. An MRI can localize the tumor to determine the best surgical approach.

The first excision provides the best chance for a cure with an aesthetic and functional outcome. For that reason careful planning, a bloodless excision, and meticulous tumor removal are imperative.

The diagnosis is confirmed by histology. When the tumor is located centrally, removal of the nail is usually necessary, but for lateral tumors, a creative approach allows for tumor removal without disruption of the nail matrix or nail bed.

Surgical success is determined by a functional and aesthetic outcome with the resolution of pain. In our case, the patient was well-healed and symptoms-free one year after surgery.

## Conflict of Interest

The authors confirm that there are no conflicts of interest.
